# Breeding indoor watercress for enhanced crop biofortification: harnessing natural variation of wild germplasm

**DOI:** 10.3389/fpls.2025.1602171

**Published:** 2025-06-20

**Authors:** Yufei Qian, Ella Katz, Daniel J. Kliebenstein, Gail Taylor

**Affiliations:** ^1^ Department of Plant Sciences, University of California, Davis, Davis, CA, United States; ^2^ Department of Genetics, Evolution and Environment, University College London, London, United Kingdom

**Keywords:** biofortification, controlled environment agriculture (CEA), glucosinolates, indoor cultivation, pre-breeding, photomorphogenesis, phenotyping, watercress (*Nasturtium officinale*)

## Abstract

We quantified the natural genetic variation of a diverse collection of wild watercress germplasm, consisting of 32 accessions collected from 16 locations in nine countries worldwide and grown in a controlled indoor environment with contrasting blue light regimes. Significant phenotypic diversity was identified for all three categories of traits: morphology and yield varied by 68% across the population (leaf size, biomass production, and stem length), with sensory (sugar content and brix), and nutritional quality (glucosinolates, vitamin C, carotenoids) varying by 45% and 43% respectively. Using two LED light regimes, control and additional blue light exposure, revealed that the watercress nutritional profile is plastic, and that the magnitude and direction of plastic responses vary depending on genotype and trait. Two glucosinolate compounds responded differently to blue light, as indolyl-3-methyl-glucosinolate increased while 4-phenylbutyl-glucosinolate decreased, but the other glucosinolate compounds tested, namely, 6-methyl-sulfinyloctyl-glucosinolate, 7-methyl-sulfinyloctyl-glucosinolate, 8-methyl-sulfinyloctyl-glucosinolate, and 2-phenylethyl-glucosinolate, showed varying responses to blue light depending on genotype. Carotenoids, especially lutein, increased consistently across the population under the additional blue light treatment, while vitamin C, glucose, and antioxidant capacity (Ferric reducing antioxidant power of plasma) all decreased after the blue light treatment. Plants were smaller and had lower biomass, but developed more leaves and branches under additional blue light. Using this phenotypic information, we identified donor germplasm lines and proposed a breeding scheme for improved nutrition and flavor alongside enhanced yield in indoor, controlled environments where there is a paucity of data. Six elite genotypes were selected that will produce a new progeny population of favorable characteristics in this powerhouse leafy-green crop.

## Highlights

- Significant genotypic diversity in the powerhouse vegetable watercress was identified through phenotyping the first global wild germplasm collection.
- Blue light exposure influenced photomorphogenesis, nutritional quality, and sensory traits, guiding the selection of elite donor germplasm for CEA breeding.

## Introduction

Watercress (*Nasturtium officinale*) belongs to the Brassicaceae family, one of the most economically important plant families, containing over 3,700 species, including vegetables, condiments, oilseeds, and fodder crops for agriculture (Warwick, 2011). Among them, watercress is a leafy green vegetable that has a long history of culinary and medicinal use (Manton, 1935). In nature, watercress is a semi-aquatic freshwater perennial, and is cultivated commercially worldwide, including but not limited to the UK, USA, Spain, Portugal, Germany, France, Iran, New Zealand, South Korea, Australia, Mexico, and China. Given its aquatic origins, watercress is highly suitable for hydroponic growth, for example, in slow-flowing streams (Hibbert et al., 2023) and in controlled environments such as indoor vertical farms (Lam et al., 2021; Qian et al., 2022, 2023). An indoor vertical farm is a controlled environment agriculture (CEA) structure, often with multiple layers in an enclosed space, in which all growth factors such as light, temperature, humidity, carbon dioxide concentration, irrigation, and nutrients are highly controlled (Kozai et al., 2019). These farms enable high-density planting and rapid, efficient harvesting in a small space and enable improved food security in many environments, including deserts, urban areas, and locations with limited natural light. The ability to alter the LED light quantity (intensity and photoperiod) and quality (wavelength) in CEA farming has enabled successful improvements in crop production compared to conventional lighting methods (Paradiso and Proietti, 2022). Plant photomorphogenesis is driven by light quality, including the red (600–700 nm) and blue (400–500 nm) parts of the spectrum (Izzo et al., 2021). Monochromatic red light promotes leaf expansion and stem elongation in addition to modulating plant reproduction, while blue light tends to control stomata dynamics, regulate whole plant size, and alter metabolism (Taíz and Zeiger, 2010). Combining light spectra of different colors has shown desirable impacts on plant yield and nutritional composition. In a recent study, Kaiser et al. (2024) found that a range of physiological and biochemical qualities, such as shelf life, aroma, and secondary metabolites, can be improved by applying additional blue light at the end of the production cycle. Lettuce plants had increased antioxidant compounds and specifically phenolics, under red light (Darko et al., 2014), whereas supplemental blue light increased lettuce leaf anthocyanin (Li and Kubota, 2009).

Watercress is recognized by the Centers for Disease Control and Prevention (CDC) as the top “powerhouse” vegetable due to its rich antioxidant profile (Di Noia, 2014). Voutsina et al. (2024) highlighted that its antioxidants include phenolic compounds, carotenoids, ascorbic acid (vitamin C), and flavonoids. Studies show that plant-derived antioxidants are linked to a lower risk of chronic diseases (Chu et al., 2002). Research indicates that watercress extract reduces reactive oxygen species (ROS) production during aerobic exercise, protecting DNA, lipids, and proteins (Fogarty et al., 2013). Additionally, various concentrations of watercress extract demonstrate anti-inflammatory properties (Sadeghi et al., 2014), protect against oxidative damage to cells (Casanova and Carballo, 2011), and detoxify lymphocyte DNA damage (Gill et al., 2007). Thus, the human health benefits of watercress consumption have been demonstrated in many peer-reviewed studies.

The distinctive pungent “peppery” taste of watercress is due to glucosinolates (GLSs), a group of sulfur-rich secondary metabolite compounds released upon wounding, mastication, or tissue damage as a plant defense mechanism (Fahey et al., 2001). When humans consume watercress, the most abundant aromatic GLS, gluconasturtiin, is hydrolyzed through the action of the enzyme myrosinase, and releases an important health-beneficial volatile, 2-phenethyl-isothiocyanate (PEITC) (Gupta et al., 2014). PEITC has been studied exclusively in clinical trials because of its antigenotoxic, antiproliferative, and antimetastatic effects on human colon cancer cell progression, especially at the initiation and invasion stages (Boyd et al., 2006). GLSs are categorized into aliphatic, indole, and aromatic types, and gluconasturtiin is an aromatic GLS found at the highest concentration in watercress (>60% of the total GLS profile) and is also found in kale and white mustard (Liang et al., 2018).

Given the antioxidant and chemopreventive benefits of watercress consumption for human health, this vegetable should be viewed as an important leafy green. However, consumers have diverse and selective taste buds and may not be willing to compromise on its peppery taste. In a panel study, the flavor of watercress was described as having a radish-like or green-vine-like flavor to a moderate level (Talavera-Bianchi et al., 2010). Customers surveyed in a study were expecting the typical pungent mouthfeel flavor of cruciferous vegetables, but the high GLS content was positively correlated with bitter and pungent tastes, and herbaceous flavors were found not to be satisfying (D’Antuono et al., 2008). Therefore, providing better-flavored watercress without compromising its natural nutrients will likely increase consumption of this healthy leafy green vegetable.

A key phase of plant breeding is the exploitation of a collection of wild relatives to discover novel traits and identify genetic variation that can be introgressed into a breeding pipeline. The use of wild relatives of many economically important crops has proven successful for the introgression of valuable traits, such as late blight (responsible for the resistance from the wild potato progenitor *Solanum demissum* (Prescott-Allen et al., 1989), and stem rust resistance from the wild wheat *Aegilops tauschii* during the Green Revolution (Kilian et al., 2010). There have also been significant successes in improving crop nutrition and quality by exploiting natural diversity. The most pertinent example was the release of Beneforté^®^ broccoli, which has 2.5–3 times higher glucoraphanin concentration than that of standard commercial cultivars, and this was achieved through genome introgression from the wild species *Brassica villosa* (Traka et al., 2013).

However, despite all the existing breeding success in a wide range of crops, these elite cultivars are generally designed to best fit conventional agricultural field production. The breeding of novel varieties specifically for the CEA environment remains limited (Folta, 2019). The breeding targets in field production greatly differ from those in CEA because the most unpredictable and changing environment is under control. Breeding priorities for CEA should be focused on fast growth cycles, easy-to-harvest plant architecture, adaptation to dense cultivation spacing, plasticity to high light intensity, and premium quality (Folta, 2019; Leong and Urano, 2018). Our aim is to screen and breed the first watercress variety specifically for the CEA environment, with outstanding nutritional and sensory traits, and, in this study, we present the first data to characterize a collection of wild-collected and grower-supplied germplasm, summarized as “wild”, for nutritional traits in CEA and its response to varying blue light treatments.

## Materials and methods

### Germplasm collection

An extensive watercress germplasm collection was used for this study. Sources of the germplasm included donations from domestic and international growers, the UK Vegetable Gene Bank (https://warwick.ac.uk/fac/sci/lifesci/wcc/genebank/), and discovery collections. These plant materials complied with all requirements for germplasm collection, testing, and verification at the time of sampling and have been described previously (Payne et al., 2015; Voutsina et al., 2016).

**Figure 1:**
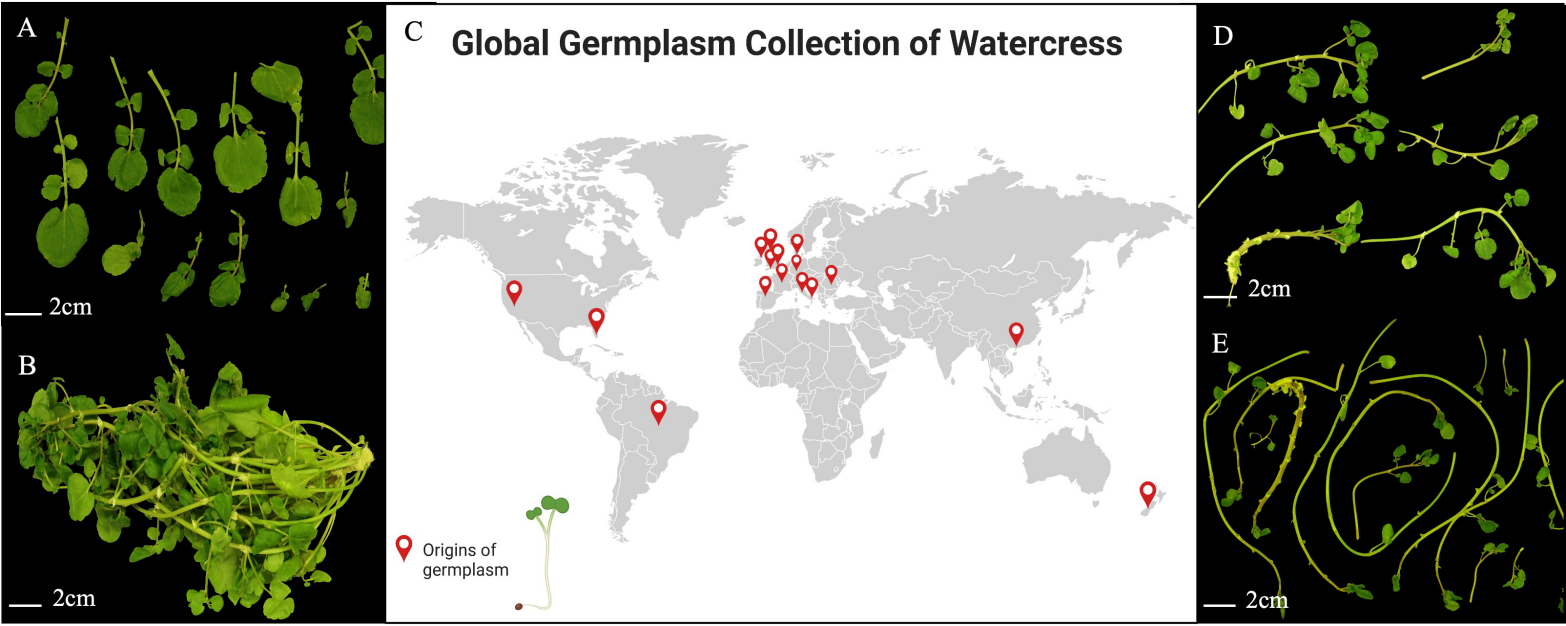
A global collection of watercress accessions, with the red pins showing the origins of the germplasm, mostly supplied by the UK Vegetable Gene Bank. **(A)** Dissection harvest of a watercress plant for measurements (leaves); **(B)** a watercress plant with high biomass; **(C)** a global map of the wild-collected accessions; **(D)** dissection harvest of a watercress plant for measurements (branches); **(E)** a watercress accession with tender and long stems and branches. The accessions used in the figure were WX0058 **(A)**, WX0059 **(B)**, WX0034 **(D)**, and WX0018 **(E)**.

In total, 32 accessions (out of 48 accessions) that passed the seed vigor test were included in this trial (for details, see **Supplementary Table 2**). The origins of these accessions are highlighted in **Figure 1**. Although both self-pollination and cross-pollination have been observed in watercress, by far the most common form of reproduction is self-pollination, and thus, overall, these lines should be considered as very likely to be “inbred”.

### Indoor vertical farm environment

The plants were grown in an indoor vertical farm facility (model: Greenery S, manufacturer: Freight Farm, MS, USA), and the LED light panels were fixed in a red-to-blue ratio of 4:1 (R4B1). The red and blue LEDs emitted light at 620–750 nm (peak at 665 nm) and 450–495 nm (peak at 455 nm), respectively. The LED light intensity under R4B1 conditions was at 220 μmol m^-2^ s^-1^ photosynthetic photon flux density (PPFD), 38 μmol m^-2^ s^-1^ under blue light only, and 187 μmol m^-2^ s^-1^ under red light only. Fertilizer containing macro- and micronutrients was dosed to maintain an electrical conductivity (EC) of 600–1000 μS. The indoor temperature was maintained between 20 and 23°C, and relative humidity was between 50 and 70% during the trial.

Control light treatment: This treatment included 18 hours of R4B1 for 21 days. The daylight integral (DLI) was 14.26 mol m^-2^ day ^-1^.

Blue Light Treatment: This treatment included 18 hours of R4B1 for 21 days, similar to the control group (DLI = 14.26 mol m^-2^ day ^-1^). During the last 7 days of the trial (from day 14 to day 21), 4 hours of additional blue light were applied (DLI = 0.54 mol m^-2^ day ^-1^) until the harvest point. These treatments were chosen as they are relevant to commercial practices, where short pulses of blue light at the end of crop production have been found to have wide-ranging impacts on crop quality and shelf-life; however, these have not been investigated for watercress (Zhou et al., 2021).

Propagation: The 32 accessions were cultivated from seeds that were directly germinated on pre-made grow media plugs of peat moss and coco coir (International Horticultural Technologies LLC, USA), along with a barcoded pot tag per seedling for record keeping. Each seed grew into a single seedling. The seedlings were transplanted into the main growth walls until their roots were established (seedlings were transplanted once root systems had visibly established within the plug) and harvested at day 21 upon transplanting.

Experiment overview: In June 2022, a trial was conducted at the vertical farm facility at the University of California, Davis. As described above, two light treatments were applied to each experimental group simultaneously. In each treatment, there were 12 biological replicates per genotype, with a total of 768 plants (12 replicates, 32 genotypes, 2 conditions) in a complete randomized block design in this trial. A two-way ANOVA was performed on all traits. Tukey’s HSD *post-hoc* tests were performed at α=0.05 to identify significantly different groups.

### Phenotyping for morphology, sensory, and nutritional traits

All plants were grown for 21 days, as described above, and were harvested for data collection. A workflow was planned to be as efficient as possible. The first activity was leaf sample collection from whole live plants for glucosinolate quantification (described below), as secondary metabolites, such as glucosinolates, are volatile and are known to degrade rapidly post-harvest. A trained team was deployed to collect leaves at the same time. The second activity was harvesting and processing 768 plants on-site for morphology and yield traits. The third activity was to snap-freeze a subset (n=256, 32*4 replicates*2 treatments) of 768 plants in liquid nitrogen, which were then stored at -80°C in a freezer and hand ground in liquid nitrogen later for wet lab assays.

### Measuring growth and plant morphology

There were 10 traits of interest that represent watercress growth, including fresh weight (FW), main stem length (Length), main stem diameter (Diameter), number of leaves (Leaves), number of branches (Branches), leaf size (Mean Leaf Area), young leaf greenness (NDVI), anthocyanin reflectance, carotenoid reflectance, and dry weight (DW) for biomass. A detailed list of these measurements can be found in **Supplementary Table 3**, including full descriptions of the 10 morphology trait measurements, harvest type, and units.

### Measuring nutritional qualities

GLS quantification: In total, 256 fresh leaf tissues (from 32 genotypes, 2 treatments, 4 replicates per treatment) were collected at the same time of day from 8 to 10 am and weighed. Then, 20–40 mg of leaf tissue was transferred into a 96-well plate and placed on dry ice. The tissues were homogenized for 3 min in a paint shaker after adding 400 μL of 90% methanol. Additional steps can be found in **Supplementary Table 3**. Individual desulfo-GLSs within each sample were separated and detected by HPLC-DAD, which were then identified, quantified by comparison to standard curves from purified compounds, and further normalized to the fresh weight.

Antioxidant capacity: The ferric-reducing antioxidant power of plasma (FRAP) quantifies the antioxidant capacity of fresh samples (Benzie and Strain, 1996). Following the protocol modified for leaf material (Payne et al., 2013), 256 snap-frozen plant samples (containing leaves and branches) were stored at -80 °C, later ground in liquid nitrogen, and measured. The full description of this protocol is in **Supplementary Table 3**.

Inductively coupled plasma mass spectrometry (ICPMS) panel: We included inductively coupled plasma mass spectrometry (Agilent 7900 ICP-MS) to examine the snap-frozen ground samples (Merieus NutriSciences, USA, licensed service provider), and the details of the elements measured can be found in **Supplementary Table 3**.

Vitamin C (ascorbic acid): Eurofins (USA) performed reverse-phase HPLC to quantify the concentration in our pooled sample (n=64, pooled by genotype in each treatment, 32*2 treatments). The detailed protocol is in **Supplementary Table 3**.

Flavonoid profiling: Service provided by Eurofins (USA). We report the concentration of Iso-quercetin, Kaempferol, Luteolin, Myricetin, Quercetin, and Rutin in pooled ground watercress tissue (**Supplementary Table 3**).

Folic acid: Analysis was performed by Eurofins (USA) using liquid chromatography (LC) with tandem mass spectrometric detection (MS/MS).

Carotenoid profiling: Service provided by Eurofins (USA). We report the carotenoid profile in total lutein, total zeaxanthin, α-cryptoxanthin, β-cryptoxanthin, lycopene (total, cis, and trans), α-carotene, and β-carotene (total, cis, and trans), from reverse-phase HPLC (**Supplementary Table 3**). Samples were pooled across genotypes by treatment.

### Measuring sensory qualities

Brix: Total soluble solids (TSSs) were measured in watercress obtained from ground snap-frozen plant tissue (n=256). The fine powder from the ground tissue was loaded into QIAshredder (QIAGEN, USA) tubes and centrifuged at 14,000 rpm for 5 minutes at 4°C to yield a crude watercress extract (as the solvent). The extract was measured in triplicate per treatment per genotype at room temperature by an Atago Digital Refractometer RX-5000i (Atago, USA).

Glucose: Glucose measurements were conducted on a One Touch Verio Flex (LifeScan Europe GmbH, Switzerland) blood glucose monitor to measure the same watercress extract prepared for Brix measurement (n=256). The range of the device used was 20 to 600 mg dL^-1^. Additional details are provided in **Supplementary Table 3**.

### Statistical analysis

All statistical analyses were performed in R (R Studio, version 1.4.1717) and in Prism (version 10.2.1). The experiment was set up in a randomized block design. Two-way ANOVA was employed on all traits, with fixed effects of light treatments, genotype, and the interaction between light and genotype. Tukey’s HSD *post-hoc* tests were performed at α=0.05 to identify significantly different groups. Principal component analysis (PCA) on the genotypic mean of each trait was also performed to reduce the dimensionality of the dataset. Multivariate analyses of the correlation matrix were performed to examine the strength and direction of all traits. All values were reported as means and standard error of the mean (SEM).

## Results

**Figure 2:**
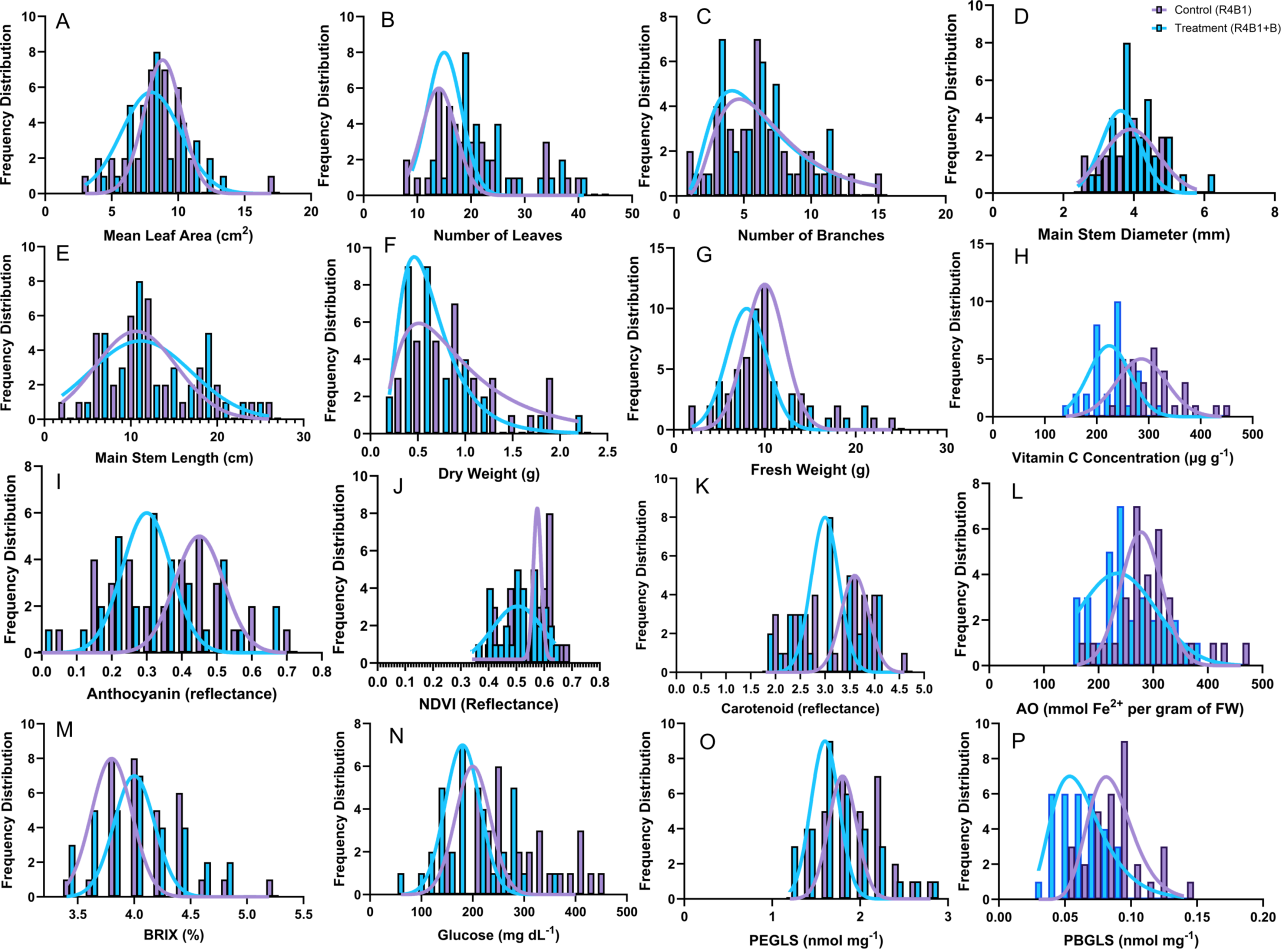
Frequency distribution of 16 traits in the two light treatments. Control (R4B1, purple) and treatment with additional blue light exposure (R4B1+B, blue). **(A)** Mean leaf area (n=314); **(B)** number of leaves (n=289); **(C)** number of branches (n=237); **(D)** main stem diameter (n=313); **(E)** main stem length (n=312); **(F)** dry weight (n=298); **(G)** fresh weight (n=570); **(H)** vitamin C concentration (n=64); **(I)** anthocyanin reflectance (n=572); **(J)** Normalized Difference Vegetation Index (NDVI, n=572); **(K)** carotenoid reflectance (n=571); **(L)** antioxidant capacity (AO, n=235); **(M)** Degree Brix (n=243); **(N)** glucose (n=235); **(O)** 2-phenylethyl-glucosinolate (PEGLS) (n=245); **(P)** 4-phenylbutyl-glucosinolate (PBGLS) (n=245).

**Figure 3:**
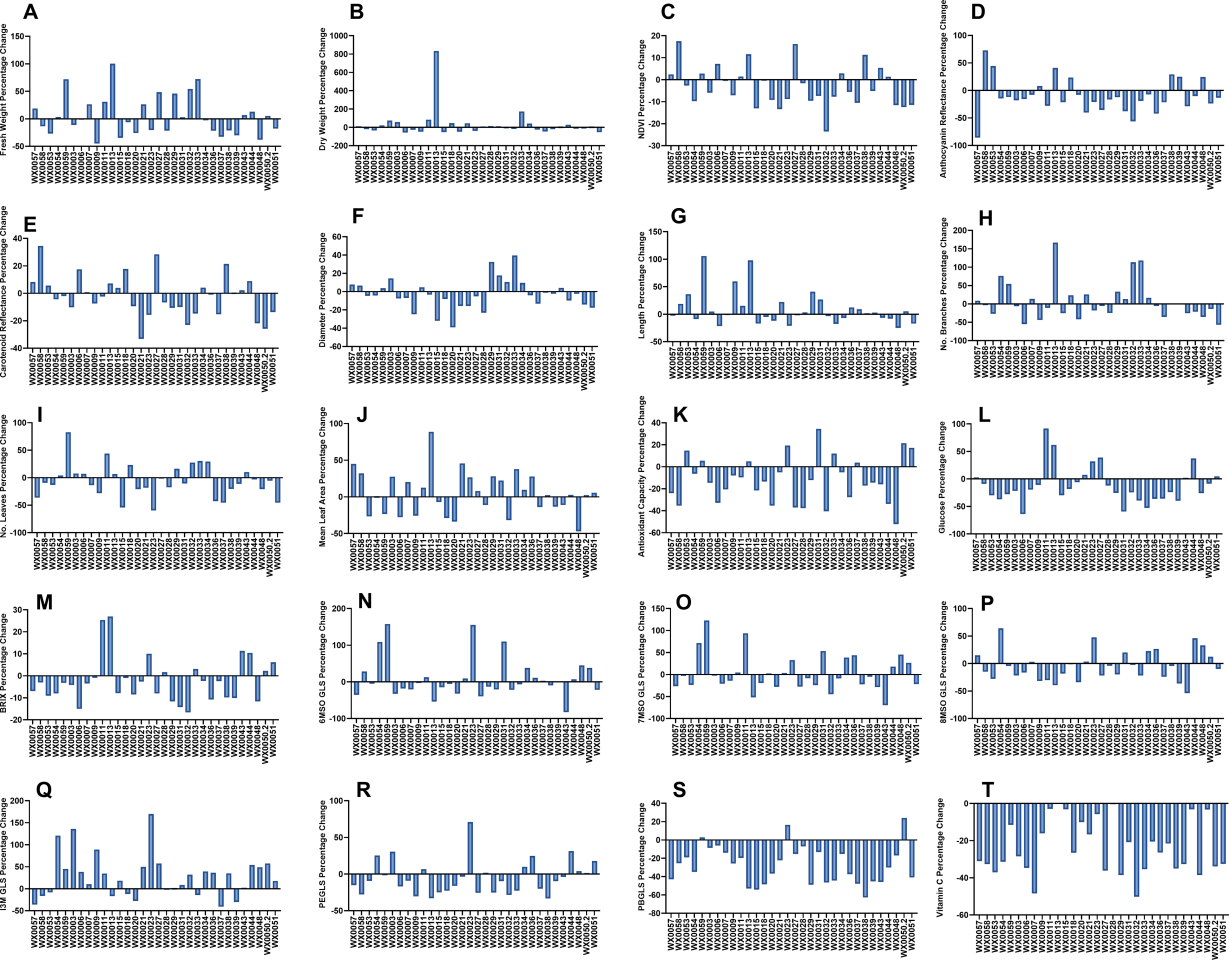
Percentage change between the control and blue light treatments for all traits from the 32 accessions, calculated as Percentage Change (%)=(treatment−control)/(control)∗100% . **(A)** Fresh weight; **(B)** dry weight; **(C)** Normalized Difference Vegetation Index (NDVI); **(D)** anthocyanin reflectance; **(E)** carotenoid reflectance; **(F)** main stem diameter; **(G)** main stem length; **(H)** number of branches; **(I)** number of leaves; **(J)** mean leaf area; **(K)** antioxidant capacity **(L)** glucose; **(M)** BRIX; **(N)** 6-methyl-sulfinyloctyl-glucosinolate (6MSO GLS); **(O)** 7-methyl-sulfinyloctyl-glucosinolate (7MSO GLS); **(P)** 8-methyl-sulfinyloctyl-glucosinolate (8MSO GLS); **(Q)** indolyl-3-methyl-glucosinolate (I3M GLS); **(R)** 2-phenylethyl-glucosinolate (PEGLS; **(S)** 4-phenylbutyl-glucosinolate (PBGLS); **(T)** vitamin C.

This global germplasm collection of watercress was found to harbor significant diversity in morphology, yield, sensory traits, and nutritional content. Since several commercial accessions were part of this study, these findings demonstrate that improvements in yield, nutrition, and sensory traits are achievable beyond what is found in current commercial watercress cultivars, especially in controlled environment systems. Of the 15 traits assessed across the whole population, and reported in **Table 1**, only one, folic acid concentration, failed to show a significant genotypic variation. Watercress genotypes showed consistent variations across control and blue light treatments (**Figure 2**), as evidenced by the two-way ANOVA, where few interactions were noted. However, large genotypic differences were often observed (**Table 1**), such that overall the trend responses were similar, but the genotypes differed in their response to the blue light treatment. A significant effect of additional blue light was observed in five of the 15 traits measured: NDVI, glucose concentration, vitamin C, FRAP, and PBGLS. Additional blue light resulted in decreased morphology and yield (p<0.05), including dry weight (control 0.84 ± 0.08 g, blue 0.72 ± 0.08 g) and fresh weight (control 9.92 ± 0.87 g, blue 9.55 ± 0.80 g), while the control light treatment resulted in plants with more branches (control 6.14 ± 0.61, blue 5.82 ± 0.49) and greater leaf number (control 19.88 ± 1.50, blue 17.51 ± 1.27). Vitamin C concentrations were reduced by exposure to the blue light treatment (control 300.19 ± 9.63 μg g^-1^, blue 223 ± 7.49 μg g^-1^), with a 26% decrease in vitamin C concentration compared to the control treatment, and this difference was consistent across genotypes. Leaf greenness, quantified as NDVI (control 0.51 ± 0.01, blue 0.50 ± 0.01), carotenoid reflectance (control 3.16 ± 0.13, blue 3.06 ± 0.12), and anthocyanin reflectance (control 0.36 ± 0.03, blue 0.33 ± 0.02), were all reduced by blue light, with the differences in the frequency distribution shown in **Figure 2**. Altering LED light regimes caused variations in the nutritional quality of watercress plants. Measurements of BRIX (control 4.12 ± 0.06°Bx, blue 4.01 ± 0.07°Bx) and glucose concentration (control 238.93 ± 14.03 mg dL^-1^, blue 198.36 ± 11.81 mg dL^-1^) indicated the same tendency of blue light exposure to result in reduced sugar content. Glucose concentration decreased by 16.8% with additional blue light. However, the response of secondary metabolites of relevance to human health showed variations in their response to blue light across genotypes in both magnitude and direction of response, as shown in **Figure 3**.

**Table 1:** Two-way ANOVA results for all traits from 32 accessions, with light treatment and genotype as the main effects. P-values between 0.05 to 0.1 are included.

	Dfs	PEGLS	NDVI (ref)	Anthocyanin (ref)	Carotenoid (ref)	BRIX	Mean Leaf Area	FW
Treatment	1	ns (p=0.09)	*	ns	ns	ns (p=0.09)	ns	ns
Genotype	31	**	****	****	****	****	****	****
Interaction	31	ns	ns	ns	ns	ns	ns	ns
		Glucose	No. of Branches	Main stem diameter	Main stem length	Folic Acid	Vitamin C	No. of Leaves
Treatment	1	***	ns	ns	ns	ns	**	ns
Genotype	31	****	****	****	****	ns	****	****
Interaction	31	ns (p=0.08)	ns	ns	ns	n/a	n/a	ns
		FRAP	PBGLS	DW	6MSO	I3M	8MSO	
Treatment	1	***	***	ns	ns	*	ns	
Genotype	31	****	****	****	****	**	****	
Interaction	31	ns	ns	ns	ns	ns	ns	

PEGLS, 2-Phenylethyl-glucosinolate; NDVI, Normalized Difference Vegetation Index; BRIX, Degrees Brix; FW, fresh weight; FRAP, ferric reducing ability of plasma; PBGLS, 4-phenylbutyl-glucosinolate; DW, dry weight; 6MSO, 6-methyl-sulfinyloctyl-glucosinolate; I3M, indolyl-3-methyl-glucosinolate; 8MSO, 8-methyl-sulfinyloctyl-glucosinolate.

ns, non-significant; *p<0.05; **p<0.01; ***p<0.001; ****p<0.0001.

**Figure 4:**
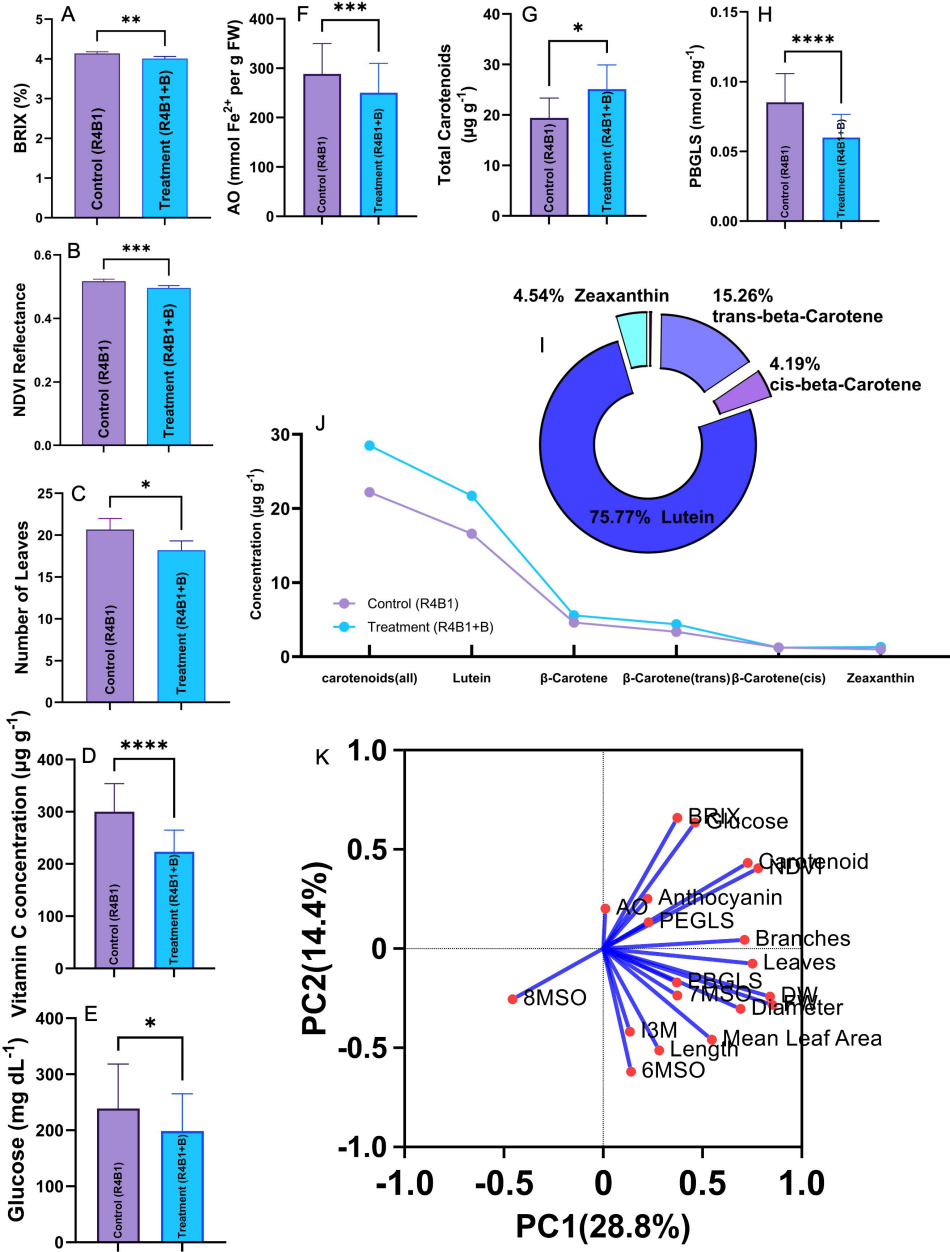
Paired t-test (two-tailed P-value, α=0.05) for nine quality traits of interest under control light treatment (Control (R4B1), purple bar) and blue light treatment (Treatment (R4B1+B), blue bar). SEM of differences are shown as “whiskers” in each figure, and the significance of the differences between means is represented as non-significant (ns); *p<0.05; **p<0.01; ***p<0.001; ****p<0.0001. **(A)** BRIX; **(B)** Normalized Difference Vegetation Index (NDVI) reflectance; **(C)** number of leaves; **(D)** vitamin C concentration; **(E)** glucose; **(F)** antioxidant capacity; **(G)** total carotenoid concentration (wet lab); **(H)** 4-phenylbutyl-glucosinolate (PBGLS). **(I)** A pie chart showing the percentage of each carotenoid compound in proportion to the total carotenoids. **(J)** Carotenoid concentration by individual compound per treatment. Samples are pooled by treatment. **(K)** Principal component analysis (PCA) of the morphological, nutritional, and sensory traits with factor loadings indicated by blue arrows. The genotypic mean of each trait was used in this analysis, and the percentage of variation explained by each principal component is included in the parentheses.

Glucosinolate concentration and composition varied considerably across the population and in response to the additional blue light treatment (**Figures 3** and **4**). In particular, there was an increase in I3M concentration by over 100% after the treatment in three accessions, WX0054, WX0003, and WX0023, while another 12 accessions increased I3M by approximately 50% under additional blue light. However, natural variation existed in this trait as nine accessions decreased in I3M, and three accessions showed little to zero percentage change in I3M after blue light exposure (**Figure 3**). In contrast to I3M, only WX0023 and WX0050.2 accessions had moderate increases in PBGLS after the blue light treatment, while the rest of the population decreased in this GLS compound (**Figure 3**). The magnitude of the response in all genotypes varied for 6MSO, 7MSO, 8MSO, and PEGLS under additional blue light. The three categories of glucosinolates, i.e., aliphatic, indole, and aromatic, were impacted by additional blue light. While I3M increased significantly across many accessions, aliphatic GLSs such as 6MSO, 7MSO, and 8MSO showed genotype-dependent responses with no overall directional trend. Aliphatic GLSs (6MSO,7MSO, and 8MSO) were insensitive to blue light. The indole I3M GLS increased by 25% (control 0.016 ± 0.001 nmol mg^-1^, blue 0.020 ± 0.001 nmol mg^-1^), while the most abundant aromatic GLS in watercress, PEGLS, also exhibited no differences between treatments (**Supplementary Figure 1D**). However, the concentration of PBGLS (**Figure 4H**), an alkyl-glucosinolate homologous to PEGLS, decreased significantly (-29.4%) under additional blue light treatment (control 0.085 ± 0.004 nmol mg^-1^, blue 0.060 ± 0.003 nmol mg^-1^). Overall, we observed that the quantity and quality of glucosinolates in watercress varied in response to blue light, but most importantly, the active GLS in watercress was not responsive to this treatment.

Total carotenoids (wet lab), including lutein, total/trans/cis beta-carotene, and zeaxanthin, were measured and significantly increased, with a 28.4% increase found (control 16.60 μg g^-1^, blue 21.70 μg g^-1^) in the watercress plants grown under additional blue light (**Figure 4J**). Lutein accounted for 75.8% of the total carotenoid profile in our watercress samples (**Figure 4I**). The flavonoid compounds detected in the watercress were quercetin (control 19.10 μg g^-1^, blue 17.30 μg g^-1^) and kaempferol (control 4.74 μg g^-1^, blue 3.68 μg g^-1^). Both chemicals slightly decreased under the blue light treatment, but this was not significant. Additionally, folic acid concentration did not differ between the treatments or across genotypes, but sample pooling for folic acid may limit the resolution of genotype-specific differences (**Table 1**). The mean concentration of the pooled samples from each treatment was 0.985 mcg.

Among the 21 traits tested across the 32 watercress accessions, genotypic differences were strongly significant in all 20 traits except for folic acid (**Table 1**). Treatment effects (**Figures 4A–H**) were significant for NDVI reflectance, glucose concentration, vitamin C concentration, FRAP, PBGLS, and I3M. Notably, we reported p-values for treatment effects between 0.05 and 0.1 for PEGLS, BRIX, and the interaction (0.05< p<0.1) between treatment and genotype in glucose concentration. Additional *post-hoc* comparisons for a few important traits can be found in **Supplementary Figure 2**.

PCA (**Figure 4K**) showed that principal component 1 (PC1), which explains 28.8% of the variation, was partially associated with secondary metabolite traits such as multiple GLS compounds. PC2, which explains 14.4% of the variation, was moderately linked to morphology traits. Carotenoid reflectance is closely related to plant yield parameters, such as fresh and dry weight, the number of leaves and branches, and NDVI. Interestingly, BRIX and glucose concentration exhibited a tight and positive correlation, and may be useful to inform future phenotyping workflows, reducing the workload of wet lab analysis.

**Figure 5:**
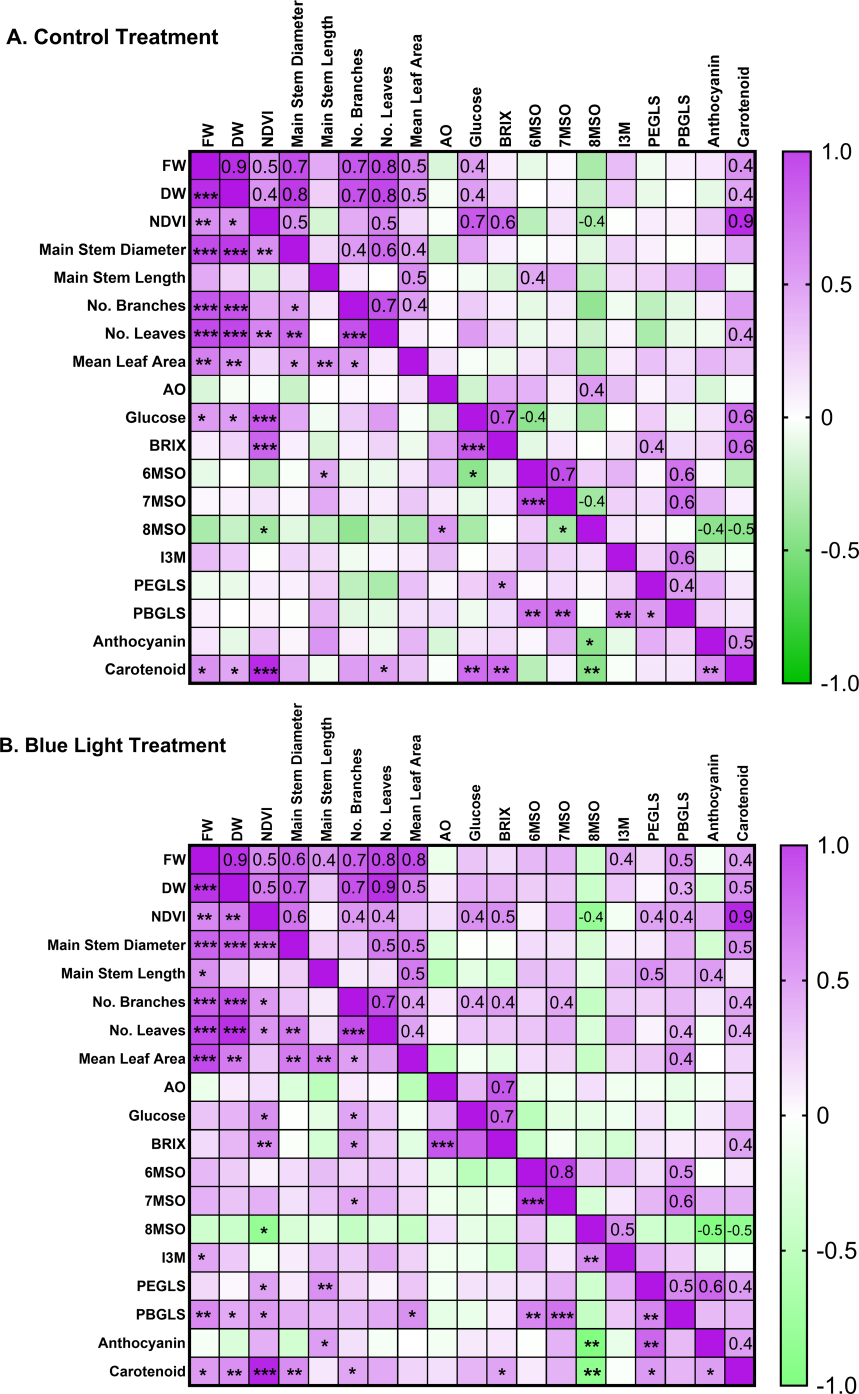
A Spearman’s correlation matrix for all traits under control light [**(A)** top] and blue light treatment p**(B)** bottom]. In each matrix, the bottom half-left triangle shows the P-values (α =0.05) indicated by an asterisk (*p<0.05; **p<0.01; ***p<0.001) of the correlation between the two variables tested. The top half-right triangle shows the correlation coefficient of the same pairs of variables, with the number indicating the strength and the signal indicating the direction. A positive correlation is denoted by the color purple and a negative correlation by the color green. Any boxes with faint colors but without an asterisk indicate a non-significant correlation.

To investigate the associations between traits in response to both treatments, a Spearman’s correlation matrix was constructed between all traits in both treatments, the control light condition (**Figure 5A**) and the additional blue light condition (**Figure 5B**). Significant positive correlations were observed consistently, pairwise, between FW, DW, NDVI, main stem diameter, number of branches, number of leaves, and mean leaf area, suggesting that robust growth patterns were observed in high-yield morphology traits. Furthermore, negative correlations were found between 8MSO and NDVI, anthocyanin, and carotenoid in both treatments, and between 7MSO and 8MSO in the control light group. Additional blue light resulted in more positive correlations, especially in plant yield traits and the entire suite of secondary metabolites. PEGLS, PBGLS, 7MSO, I3M, and anthocyanin were all found to be positively correlated with various morphological traits under the additional blue light (**Figure 5B**), while these correlations were not significant under the control light treatment (**Figure 5A**).

There were several pairs of correlations that remained consistent across treatments. Consistent correlations in a Spearman’s matrix tend to establish relationships between two quantitative variables measured in a positive or negative direction, with various magnitudes. For example, NDVI and carotenoid reflectance were strongly correlated (+0.7 in the control group, +0.9 in the blue group). Carotenoid reflectance and anthocyanin reflectance had medium correlations (+0.5 in the control group, +0.4 in the blue group). 8MSO negatively correlated with NDVI (-0.4 in both treatments), anthocyanin reflectance (-0.4 in the control group, -0.5 in the blue group), and carotenoid reflectance (-0.5 in both treatments). As shown above in **Figure 4** PCA, we noticed the same strong positive correlation between BRIX and glucose (+0.7 in both treatments). These consistent correlations indicated important groupings of traits. For example, in **Figure 5**, a high NDVI value is an indicator of more chlorophyll production, which leads to better photosynthesis activities and greater fresh weight (consistent strong positive correlations under both treatments). Similarly, main stem diameter positively correlated with the number of leaves (+0.6 in the control group, +0.5 in the blue group), suggesting that robust growth of main stems leads to higher leaf yield. However, 8MSO correlated negatively with NDVI, anthocyanin, and carotenoids in both treatments, suggesting trade-offs in energy allocation during the production of secondary metabolites, antioxidants, and green tissue in plants. Overall, these data highlight the biological significance of these correlations, where traits underpinning yield and quality have been determined. These findings are novel, as there is a scarcity of literature specifically addressing watercress.

The blue light treatment reversed the following correlations between traits. 8MSO had a negative correlation (-0.4) with 7MSO under control light conditions, but this was found to be non-significant under the blue light. Antioxidant capacity did not have a significant correlation with BRIX under control light conditions, but did so after additional blue light.

**Figure 6:**
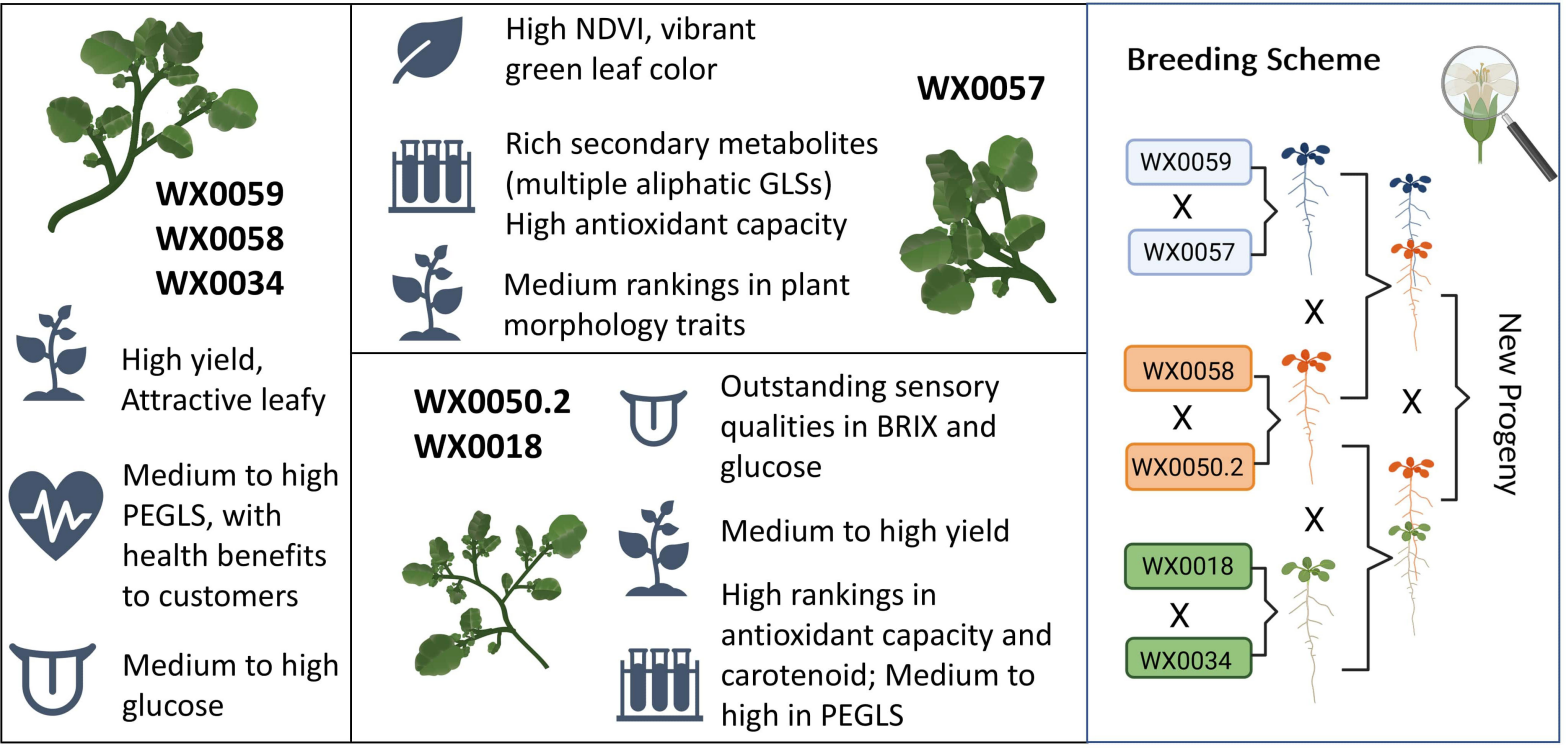
A description of watercress ideotypes from the wild population. Selected accessions possess at least one highly ranked trait from one of the three categories: yield, sensory, and nutritional quality. We propose a future breeding scheme that will produce a new progeny population of favorable yield and nutritional characteristics for consumers. “X” stands for crosses between the parental accessions. Pollination of watercress is manually performed, usually at dawn, under a magnifying glass, as shown in the upper right corner of the panel.

Out of the 32 accessions in the trial, we identified six genotypes that were informative as ideotypes for a future breeding program (**Figure 6**). We defined the ideotypes based on a combination of high total biomass and NDVI, nutritional enhancement including carotenoids, GLSs, and antioxidant capacity, and favorable sensory attributes such as medium to high BRIX and glucose values. We selected accessions that exhibited at least one outstanding category to create “good x good” crosses. The discovery of diverse traits through pre-breeding screening will greatly increase the efficiency of introgression of desirable traits to accelerate the future breeding of watercress for enhanced nutrition (**Figure 6**). WX0059, WX0058, and WX0034 all exhibited high yield, biomass, and number of leaves in both treatments. These three accessions were also ranked medium to high in nutritional and sensory qualities at the same time. WX0057 had a vibrant green color. This color represents a popular physical appearance to customers. It contained high concentrations of multiple aliphatic GLSs and antioxidants (high FRAP measurements), which are highly associated with human health benefits. To avoid the common bitterness and grassy taste in leafy greens, we also selected accessions WX0050.2 and WX0018 for their consistent and outstanding sensory rankings in BRIX value and glucose concentration. Furthermore, both accessions possessed good secondary metabolite profiles and medium-to-high yields. Future informed watercress breeding programs may utilize these elite donor lines in new population development and marker-assisted selection with genotyping-by-sequencing (GBS) strategies. In combination with ongoing work with genotyping, these data will be highly informative in developing genomic selection and modeling strategies.

## Discussion

Watercress is an understudied leafy green crop that contains important nutrients that benefit human health (Gupta et al., 2014). The CDC ranked watercress first among “powerhouse vegetables”, with a better nutrient density than kale, spinach, and lettuce (Di Noia, 2014). For the first time, this study establishes foundational knowledge on indoor-grown watercress by presenting the first systematic evaluation of trait variation in indoor-grown watercress from a global wild germplasm collection. Our results identified abundant natural diversity to be exploited in breeding and selection. We also quantified the effects of two LED light regimes on this unique watercress population and described it in terms of plant yield, morphology, sensory qualities, and nutritional content. Using this information, we identified new ideotypes for the CEA market that have the potential to be considered “good x good” crosses for future commercialization. Genotypic effects within the population were significant for all traits measured, except for folic acid. Blue light effects vary. Notably, the concentration of each GLS compound responded in polarized ways to additional blue light, as I3M increased, while PBGLS decreased significantly. We reported a consistent reduction in vitamin C within the population after the treatment with blue light. Total carotenoids, specifically lutein, in contrast, increased following the modest blue light treatment. The biological significance of a genotype-by-blue-light interaction is subject to interpretation. Using *Arabidopsis* as a model system, plants respond to blue light by activating different blue-light photoreceptors, i.e., cryptochrome 1, cryptochrome 2, and phototropin (Keuskamp et al., 2011; Lin, 2000). It seems likely that these photoreceptors or their consequential signal transduction pathways are differentially expressed across our watercress collection, which may reflect their environments of origin and adaptation to varying light and stress conditions. Taken together, these results suggest there is significant potential to select and breed watercress with improved yield and better nutritional content for the indoor controlled environment market.

The narrow genetic base of released cultivars has been a common challenge in a wide range of horticultural and agronomical crops, including but not limited to wheat, lettuce, melon, and legumes (Dempewolf et al., 2017). Regardless of the genetic and genomic tools, natural plant genetic resources (germplasm) are the basis for exploiting new and diverse genetic variation, which is critical for crop improvement (Diepenbrock and Gore, 2015). There have been multiple cases of introgressed beneficial traits from wild relatives, including for yield, morphology, adaptability, resilience, and biofortification (Prescott-Allen et al., 1989). One of the most prominent cases is the uninterrupted discovery of over 40 disease resistance genes found in tomato wild relatives since 1982 (Rick and Chetelat, 1995), alongside the use of wild types to improve tomato fruit quality for desirable consumption, including soluble solid content, fruit size and color, and postharvest preservation (Tanksley and McCouch, 1997). Given the advantages of utilizing wild relatives in breeding, our 32 watercress accessions, collected from geographically distinct locations, showed valuable, strong genotypic effects in the two-way ANOVA table (**Table 1**) on morphology, biochemistry, and sensory traits. We captured a wide range of natural variation in plant yield traits and morphology in the wild population. Across the 32 accessions, plant fresh weight ranged from 3.2g to over 20g; the number of leaves ranged from 8 to 40 leaves per plant; the number of branches varied between 1 to 15 per plant.

The Brassicaceae family includes many economically important vegetables, such as broccoli, cabbage, Brussels sprouts, kale, radish, and Bok choy, and this family is well-known for containing abundant phytonutriceuticals, such as glucosinolate compounds (Ishida et al., 2014). Consumption of Brassicaceae vegetables is recommended because they are rich sources of health-improving phytochemicals that reduce the risk of cancer and chronic diseases (Chu et al., 2002). GLSs are a group of sulfur-containing plant secondary metabolites, which are categorized as aliphatic, indolic, or aromatic. Among 12 common Brassicaceae vegetables, watercress was found to have the highest concentration of the aromatic GLS gluconasturtiin (>60% of the total GLS profile) (Liang et al., 2018). The hydrolysis product of gluconasturtiin converts to PEITC, which has been proven to have positive tumor-modulatory effects in human cancer cells (Boyd et al., 2006). Watercress contains not only GLSs but also has a rich profile of polyphenolic compounds (anthocyanins), phenolics (flavonoids), carotenoids, B-vitamin family (folic acid), and vitamin C (as ascorbic acid) (Voutsina et al., 2024), which we have quantified in this study.

The Indole I3M GLS in watercress increased by 25% with the additional blue light treatment, in agreement with Wang et al. (2022), who also observed a significant increase in a hydrolysis product (indole-3-carbinol, I3C) of I3M GLS in broccoli sprouts under the monochromatic blue LED light at 200 μmol m^-2^ s^-1^. In contrast to the research reported here, it was previously shown that aliphatic GLSs tend to increase under various blue light intensities (Carvalho and Folta, 2014; Xue et al., 2021), as an abiotic stress response mechanism for photoprotection (Landi et al., 2015; Ramakrishna and Ravishankar, 2011). Our experiment did not reveal an increase in aliphatic GLS, and this may be due to the species differences in GLS composition and their differential response to blue light. The most abundant aromatic GLS in watercress is PEGLS, and its concentration did not differ between treatments (p=0.09). However, PBGLS, a glucosinolate with two additional carbons in the carbon-chain in comparison to PEGLS, was not found in watercress growing in outdoor field conditions, but was first detected in plants growing indoors under different ratios of red and blue LED light (Qian et al., 2022) and was significantly reduced after additional blue light in this experiment. There are very few published data that focus on PBGLS (which does not have a common name). Other than in watercress, this alkyl-benzyl compound has also been found in *Diplotaxis*, *Eruca*, and *Armoracia* (horseradish) plants (D’Antuono et al., 2009; Dekić et al., 2017). PBGLS possesses antimicrobial capacity (Dekić et al., 2017). Currently, the biochemical mechanism resulting in reduced PBGLS under additional blue light in watercress, as observed here, remains unknown.

Carotenoids are a large group of pigments distributed in photosynthetic and non-photosynthetic plant tissues. Carotenoid concentrations in watercress exhibit a strong association with genotype and fertilizer application. Kopsell et al. (2007) found a boost of β-carotene, lutein, and zeaxanthin with increased nitrogen fertilizer application. In our study, the results show that 12 accessions had a greater carotenoid reflectance value (indicating more carotenoids) after the blue light treatment, varying from 5% to over 35% based on genotype. However, the relative abundance of each carotenoid compound influences crop color, with lutein being a more light-yellow color, and beta carotene and zeaxanthin associated with a deeper orange color (LaPorte et al., 2022; Yuan et al., 2015). In leafy green vegetables, the major carotenoids are lutein and beta carotene, as was found here for watercress. We conducted both a carotenoid reflectance measurement and a wet lab assay using the same leaf tissue, but the results from these two test methods were inconsistent. Carotenoid concentrations were lower in the blue light treatment as measured by reflectance; however, the wet lab HPLC results concluded that blue light significantly increased total carotenoids, especially lutein, in watercress. Handheld proximal spectral reflectance devices may have limitations in accurately dissecting each carotenoid compound when measuring its reflectance, while a state-of-the-art HPLC enables the full profiling of chemicals. Our results show that 76% of the carotenoids in watercress were lutein, a light-yellow pigment, and its concentrations increased in additional blue light by 28.4%. Similarly, Samuolienė et al. (2017) found that lutein concentration in three microgreens all increased under blue light, especially in mustard, also a Brassicaceae leafy green, which responded well to medium-high intensities of blue light by increasing lutein concentration. Lutein is a critical bioactive chemical that has important health benefits to human retinal function (Kijlstra et al., 2012). Therefore, our findings highlight a mechanism that blue light management can be utilized in future plant productions to enhance the nutritional quality of CEA grown crops.

The ascorbic acid concentration (mainly as vitamin C) in watercress plants grown under the control light treatment (R4B1) was significantly higher than with the additional blue light treatment (R4B1+B). WX0007 and WX0032 had the greatest reduction in vitamin C concentration after the treatment, both more than 40%, followed by 14 accessions with at least a 20% decrease. Only WX0011, WX0015, WX0028, WX00043, and WX0048 responded to the additional blue light by showing minimal or no response in this trait. This observed phenomenon contrasted with some studies on the effect of blue LED light on crops, but the majority of the light treatments in the literature were only under monochromatic bandwidth alone (RGB). Kang et al. (2020) found blue light irradiation for 5 days increased the vitamin C content in Chinese cabbage seedlings by activating the biosynthesis pathways of ascorbic acid. However, in our study, there may have been a synergistic effect of a mixture of red and blue light that promoted vitamin C accumulation (Bian et al., 2015). Light intensity plays a significant role in vitamin C concentration in plants. High light intensity increases vitamin C accumulation compared to low light (Foyer et al., 2020). Li et al. (2019) and Zheng et al. (2018) tested four different intensities (0, 50, 100, and 150 μmol m^-2^ s^-1^) on two Brassica vegetables and concluded that supplemental blue light intensity at 50 and 100 μmol m^-2^ s^-1^ yielded the best vitamin C quality. However, due to the limitation of our facility, the blue light alone (38 μmol m^-2^ s^-1^) had a lower intensity than the “low” treatment in the above-described experiments. This finding highlights a research gap to optimize lower light intensity and spectral composition in controlled environments to enhance vitamin C retention. Additionally, vitamin C synthesis is closely related to the plant’s developmental stage (Attolico and De Tullio, 2006), diurnal pattern (Dutilleul et al., 2003), and alternating intervals of red and light irradiation (Chen et al., 2017).

## Conclusion

We identified significant variation in growth, sensory, and nutritional traits in a globally diverse wild germplasm collection of watercress in an indoor vertical farm with two different LED light regimes (control: R4B1 and treatment: R4B1+Blue). We found that WX0058 and WX0034 had more than a 108.5% increase in yield relative to current commercial genotypes. Similarly, the nutritional traits of the wild population increased by 28.9% (PEGLS), 83.3% (I3M), 131% (8MSO), 82.1% (AO), and 31.9% (Glucose) (relative to commercially available genotypes).

Under additional blue light, the watercress plants developed fewer leaves and branches, had increased I3M GLS and carotenoids, especially lutein, but had reduced biomass, PBGLS, vitamin C, glucose, and total antioxidants (FRAP). The rest of the traits exhibited varying responses to the treatment depending on genotypes. Given the current limited number of new crop varieties for controlled environments, this pre-breeding trial identified novel traits of wild relatives of watercress, providing six elite donor lines for future CEA breeding.

## Data Availability

The datasets presented in this study can be found in online repositories. The names of the repository/repositories and accession number(s) can be found below: https://doi.org/10.5061/dryad.xsj3tx9qw.
