# Supplementary material for: Breeding indoor watercress for enhanced crop biofortification: harnessing natural variation of wild germplasm
Source: Front Plant Sci. 2025 Jun 20;16:1602171. doi: 10.3389/fpls.2025.1602171 (PMC12226469; doi:10.3389/fpls.2025.1602171)
Supplement: Supplementary file 1 [file Table1.docx]

**Supplemental Table 3. List of measurements of all morphology traits.** Abbreviations are in bold. Fresh weight, **FW**. Main stem length, **Length**. Main stem diameter, **Diameter**. Number of leaves, **Leaves**. Number of branches, **Branches**. Leaf size, **Mean Leaf Area**. Young leaf greenness, **NDVI,** the normalized difference vegetation index. Dry weight, **DW**.

|  | Destructive Harvest | Description | Unit |
| --- | --- | --- | --- |
| Fresh weight (FW) | Yes | Weight individual plant on-site at the harvest point. | g |
| Main Stem length  (Length) | Yes | Measure the main stem from the base to the tip. | cm |
| Main Stem diameter (Diameter) | Yes | Measure at the midpoint of the main stem | mm |
| Number of leaves  (Leaves) | Yes | Layout all the leaves on a chopping board | Count number |
| Number of branches  (Branches) | Yes | Layout all the branches on a chopping board | Count number |
| Leaf size (Mean Leaf Area) | Yes | Layout all the leaves and leaflets on a chopping board with white background and take a picture. Use a ruler to scale the background |  |
| Young Leaf greenness  (NDVI) | No | Perform NDVI measurements on the 6^th^ leaflet from the innermost rosette | Band ratio |
| Anthocyanin reflectance | No | Perform anthocyanin measurements on the 6^th^ leaflet from the innermost rosette | Band ratio |
| Carotenoid reflectance | No | Perform carotenoid measurements on the 6^th^ leaflet from the innermost rosette | Band ratio |
| Dry weight (DW) | Yes | Paper bag individual plant and put in the dryer at 80 °C for 48 hours. | Gram |

| **Nutritional Quality measurements** | | |
| --- | --- | --- |
| Carotenoid profiling | We report the carotenoid Profile in Total Lutein, Total Zeaxanthin, a-Cryptoxanthin, b-Cryptoxanthin, Lycopene (Total, cis and trans), a-Carotene, b-Carotene (Total, cis and trans), from reverse-phase HPLC and employed an internal standard for serum-based matrices and an external standard based on peak areas for all other types of samples (Eurofins, USA). The HPLC is equipped with either scanning UV or UV/DAD detection. The separation uses a C18 column with Ti frits. The mobile phase is a mixture of acetonitrile, p-dioxane, methanol, isopropanol, triethylamine, and ammonium acetate. Carotenoids are detected at 450 nm in the visible spectrum. Fresh plant tissue was snap frozen and ground in liquid nitrogen before use. | |
| Flavonoids profiling | We report the concentration of Iso-quercetin, Kaempferol, Luteolin, Myricetin, Quercetin, and Rutin in fresh ground watercress tissue. The method uses gradient reversed-phase HPLC with UV and optionally coulometric detection. The method scans UV from 240 nm to 370 nm. Components are detected at 260, 275, and 330 nm. Eurofins (USA) provided the service. | |
| Folic Acid | Samples were extracted by agitation using an acidic aqueous-solvent mixture, followed by the addition of a small amount of alkaline solution to precipitate proteins and aid in chromatography. Analysis was performed by Eurofins (USA) using liquid chromatography (LC) with tandem mass spectrometric detection (MS/MS). | |
| Vitamin C (Ascorbic Acid) | Eurofins (USA) performed reverse-phase HPLC. The method is calibrated daily with neat standards that are assigned concentrations using both gravimetric values and the molar extinction coefficient. The concentrations are corrected for HPLC purity. The calibration method is based on external standard using peak areas. We measured vitamin C using UV at 245 nm and electrochemically at 600 mV. The method is based upon reduction with TCEP in an aqueous buffer followed by acidic extraction. Samples are separated using a C18 column without ion-pairing. | |
| ICPMS Panel | We included the inductively coupled plasma mass spectrometry (ICP-MS) to snap frozen ground samples (Merieus NutriSciences, USA). The list of elements measured at trace levels were Aluminum, Antimony, Arsenic, Barium, Beryllium, Bismuth, Boron, Cadmium, Calcium, Chromium, Cobalt, Copper, Iron, Lead, Lithium, Magnesium, Manganese, Mercury, Molybdenum, Nickel, Phosphorus, Potassium, Selenium, Silver, Sodium, Strontium, Thallium, Thorium, Tin, Titanium, Uranium, Vanadium, Zinc, and Zirconium. | |
| Antioxidant Capacity | Ferric reducing antioxidant power of plasma (FRAP) quantifies the antioxidant capacity of fresh samples (Benzie & Strain, 1996). The snap-frozen sample was stored in -80 ⁰C, and later ground in liquid nitrogen. Plant sap was extracted using QIAshredder (QIAGEN) tube, centrifuged at 14,000 rpm for 5 minutes at 4⁰ C. Then, two-fold serial dilutions of iron sulphate heptahydrate with the concentration from 0.25 mmol to 8 mmol were added along with FRAP reagent mix. Absorbance was read at 584 nm on a spectrophotometer, as described previously for watercress (Payne et al., 2013). | |
| Glucosinolates (GLSs) Quantification | Tissues were homogenized for 3 min in a paint shaker after adding 400 ul of 90% methanol, centrifuged, and the supernatants were transferred to a 96-well filter plate with DEAE sephadex. Filter plate with DEAE sephadex was washed once with water, 90% methanol, and water again. The sephadex-bound GLS were eluted after an overnight incubation with 110 μL of sulfatase. Individual desulfo-GLSs within each sample were separated and detected by HPLC-DAD, identified, quantified by comparison to standard curves from purified compounds, and further normalized to the fresh weight (Kliebenstein et al., 2001; Qian et al., 2023). | |
| **Sensory Quality measurements** | | |
| Brix | | Total soluble solids (TSS) were measured in watercress obtained from ground snap frozen whole plant tissue. Fine powder of the ground tissue was loaded into QIAshredder (QIAGEN) tubes, centrifuged at 14,000 rpm for 5 minutes at 4⁰ C to yield a crude watercress extract (as the solvent). The extract was measured in triplicates per treatment per genotype at room temperature by an Atago Digital Refractometer RX-5000i (Atago, USA). |
| Glucose | | Glucose measurements were conducted on a One Touch Verio Flex (LifeScan Europe GmbH, Switzerland) blood glucose monitor to measure the same watercress extract prepared for Brix measurement. The range of the device used was 20 to 600 mg dL^-1^. The same protocol was used to measure lettuce leaf glucose content (Hernandez et al., 2020). |

Benzie, I. F. F., & Strain, J. J. (1996). The ferric reducing ability of plasma (FRAP) as a measure of “antioxidant power”: The FRAP assay. *Analytical Biochemistry*. https://doi.org/10.1006/abio.1996.0292

Hernandez, E., Timmons, M. B., & Mattson, N. S. (2020). Quality, yield, and biomass efficacy of several hydroponic lettuce (Lactuca sativa l.) cultivars in response to high pressure sodium lights or light emitting diodes for greenhouse supplemental lighting. *Horticulturae*, *6*(1). https://doi.org/10.3390/horticulturae6010007

Kliebenstein, D. J., Lambrix, V. M., Reichelt, M., Gershenzon, J., & Mitchell-Olds, T. (2001). Gene duplication in the diversification of secondary metabolism: Tandem 2-oxoglutarate-dependent dioxygenases control glucosinolate biosynthesis in arabidopsis. *Plant Cell*, *13*(3). https://doi.org/10.1105/tpc.13.3.681

Payne, A. C., Mazzer, A., Clarkson, G. J. J., & Taylor, G. (2013). Antioxidant assays - consistent findings from FRAP and ORAC reveal a negative impact of organic cultivation on antioxidant potential in spinach but not watercress or rocket leaves. *Food Science & Nutrition*. https://doi.org/10.1002/fsn3.71

Qian, Y., Hibbert, L. E., Katz, E., Smith, H. K., Kliebenstein, D. J., & Taylor, G. (2023). Watercress yield and quality vary depending on both genotype and environment: Results from highly contrasting growing systems of California and UK. *Scientia Horticulturae*, *319*. https://doi.org/10.1016/j.scienta.2023.112154
