# Supplementary material for: Breeding indoor watercress for enhanced crop biofortification: harnessing natural variation of wild germplasm
Source: Front Plant Sci. 2025 Jun 20;16:1602171. doi: 10.3389/fpls.2025.1602171 (PMC12226469; doi:10.3389/fpls.2025.1602171)
Supplement: Supplementary file 2 [file Table2.docx]

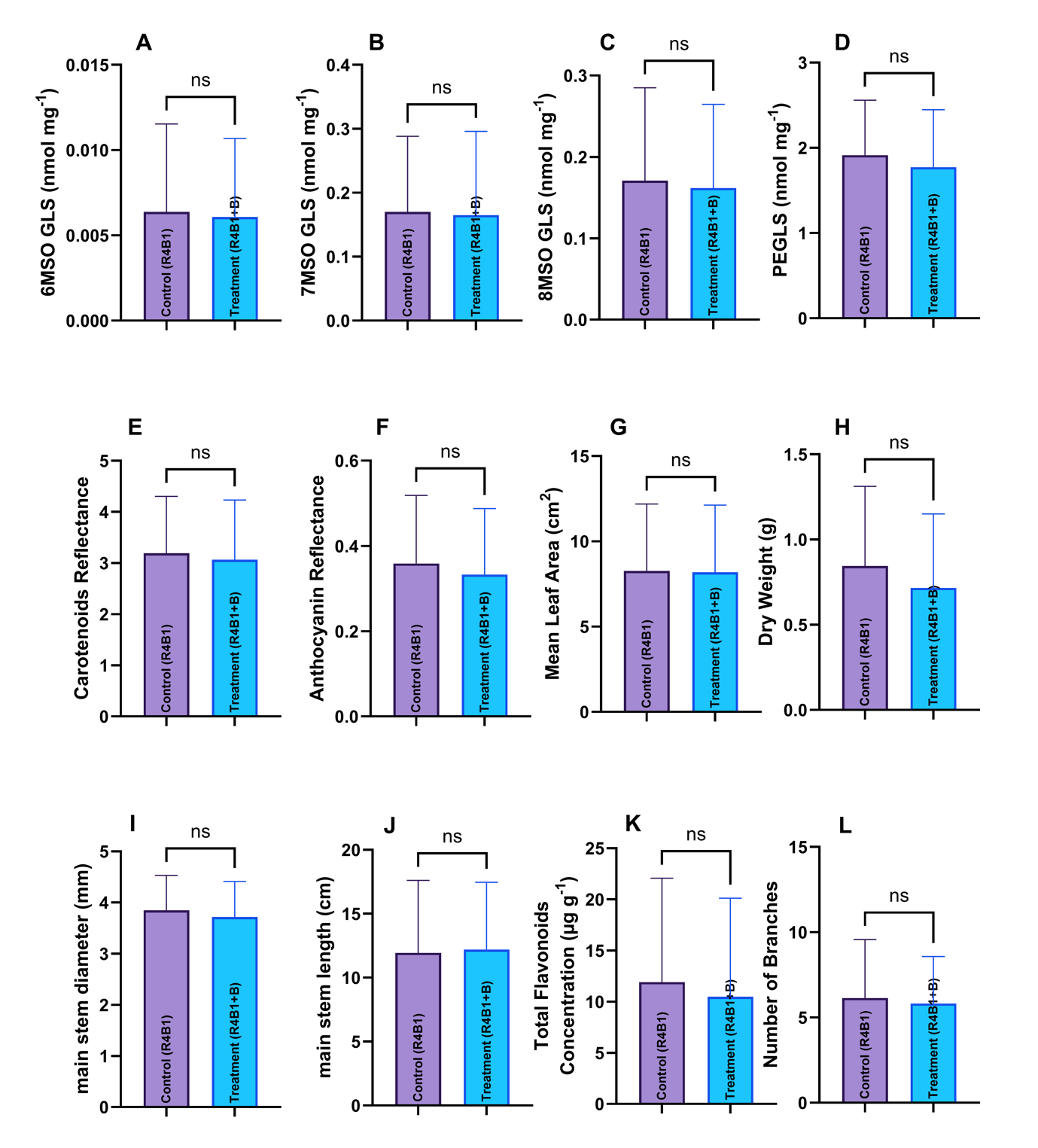


**Supplemental Figure 1.** Paired t test (two tailed P value, α=0.05) of 12 traits with non-significant results. SEM of differences were shown as “whiskers” in each figure, and the significance of the differences between means were represented as ns= non-significant. A.6MSO B.7MSO C.8MSO D. PEGLS E. Carotenoids Reflectance F. Anthocyanin Reflectance G. Mean Leaf Area H. Dry Weight I. Main Stem Diameter J. Main Stem Length K. Total Flavonoids L. Number of Branches. Control light treatment (Control (R4B1), purple bar) and blue light treatment (Treatment (R4B1+B), blue bar.
