# Supplementary material for: Breeding indoor watercress for enhanced crop biofortification: harnessing natural variation of wild germplasm
Source: Front Plant Sci. 2025 Jun 20;16:1602171. doi: 10.3389/fpls.2025.1602171 (PMC12226469; doi:10.3389/fpls.2025.1602171)
Supplement: Supplementary file 3 [file Table3.docx]

**
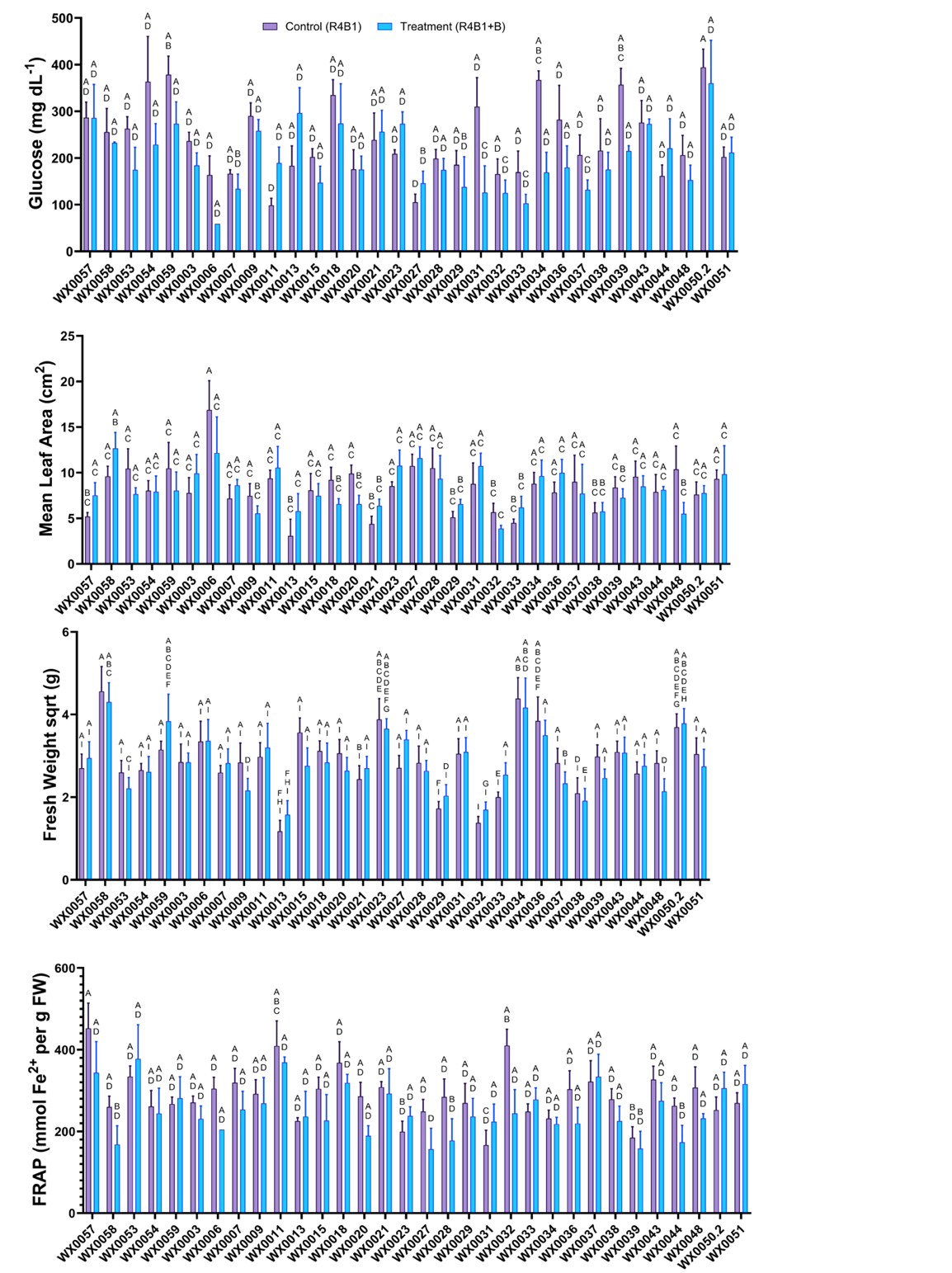
**

D

C

B

A

**Supplemental Figure 2.** Genotypic mean comparison of four traits with SEM. Post-hoc Tukey’s multiple comparison (alpha=0.05) indicated by mean separation letters. Control light treatment, purple bar) and blue light treatment, blue bar. A. Glucose B. Mean Leaf Area C. Fresh Weight D. FRAP
