# Supplementary material for: Breeding indoor watercress for enhanced crop biofortification: harnessing natural variation of wild germplasm
Source: Front Plant Sci. 2025 Jun 20;16:1602171. doi: 10.3389/fpls.2025.1602171 (PMC12226469; doi:10.3389/fpls.2025.1602171)
Supplement: Supplementary file 4 [file Table4.pdf]

Supplementary Table 1. Two-way ANOVA results for 20 traits reported

Traits	SS	DF	MS	F (DFn, DFd)	P value
**PEGLS**					
Interaction	10.6	31	0.342	F (31, 181) = 0.8831	P=0.6477
genotype	23.74	31	0.766	F (31, 181) = 1.978	P=0.0031
treatment	1.117	1	1.117	F (1, 181) = 2.883	P=0.0912
**NDVI**					
Interaction	0.284	31	0.009	F (31, 508) = 0.8928	P=0.6360
genotype	2.885	31	0.093	F (31, 508) = 9.067	P<0.0001
treatment	0.04399	1	0.044	F (1, 508) = 4.287	P=0.0389
**Anthocyanin (ref)**					
Interaction	1.176	31	0.038	F (31, 508) = 0.9305	P=0.5771
genotype	12.59	31	0.406	F (31, 508) = 9.963	P<0.0001
treatment	0.09243	1	0.092	F (1, 508) = 2.267	P=0.1328
**Carotenoid (ref)**					
Interaction	32.25	31	1.04	F (31, 507) = 1.099	P=0.3285
genotype	226.1	31	7.294	F (31, 507) = 7.708	P<0.0001
treatment	1.412	1	1.412	F (1, 507) = 1.492	P=0.2225
**BRIX**					
Interaction	7.359	31	0.237	F (31, 179) = 1.069	P=0.3785
genotype	24.04	31	0.775	F (31, 179) = 3.493	P<0.0001
treatment	0.6593	1	0.659	F (1, 179) = 2.970	P=0.0866
**Mean Leaf Area**					
Interaction	362	31	11.68	F (31, 250) = 0.9135	P=0.6029
genotype	1257	31	40.55	F (31, 250) = 3.172	P<0.0001
treatment	0.5036	1	0.504	F (1, 250) = 0.03939	P=0.8428
**FW**					
Interaction	20.93	31	0.675	F (31, 506) = 0.5749	P=0.9694
genotype	245.3	31	7.912	F (31, 506) = 6.738	P<0.0001
treatment	0.4351	1	0.435	F (1, 506) = 0.3705	P=0.5430
**Glucose**					
Interaction	285620	31	9214	F (31, 171) = 1.427	P=0.0806
genotype	824401	31	26594	F (31, 171) = 4.120	P<0.0001
treatment	91117	1	91117	F (1, 171) = 14.12	P=0.0002
**Number of Branches**					
Interaction	278.7	31	8.989	F (31, 173) = 0.5886	P=0.9587
genotype	1985	31	64.02	F (31, 173) = 4.192	P<0.0001
treatment	5.421	1	5.421	F (1, 173) = 0.3550	P=0.5521
**Main Stem Diameter**					

Interaction	25.42	31	0.82	F (31, 249) = 0.9074	P=0.6122
genotype	105.8	31	3.412	F (31, 249) = 3.775	P<0.0001
treatment	1.257	1	1.257	F (1, 249) = 1.391	P=0.2394
**Main Stem Length**					
Interaction	502	31	16.19	F (31, 248) = 0.6904	P=0.8919
genotype	8261	31	266.5	F (31, 248) = 11.36	P<0.0001
treatment	4.889	1	4.889	F (1, 248) = 0.2084	P=0.6484
**Folic Acid**					
genotype	0.000979	7	1E-04	F (7, 2) = 3.832	P=0.2225
treatment	0.000096	1	1E-04	F (1, 2) = 2.630	P=0.2463
**Vitamin C**					
genotype	100291	30	3343	F (30, 30) = 2.625	P=0.0050
treatment	92362	1	92362	F (1, 30) = 72.51	P<0.0001
**Number of Leaves**					
Interaction	1.18	31	0.038	F (31, 225) = 0.6093	P=0.9499
genotype	5.119	31	0.165	F (31, 225) = 2.643	P<0.0001
treatment	0.1141	1	0.114	F (1, 225) = 1.825	P=0.1780
**FRAP**					
Interaction	178693	31	5764	F (31, 171) = 0.8553	P=0.6880
genotype	658851	31	21253	F (31, 171) = 3.153	P<0.0001
treatment	79167	1	79167	F (1, 171) = 11.75	P=0.0008
**PBGLS**					
Interaction	0.02491	31	8E-04	F (31, 181) = 1.017	P=0.4499
genotype	0.056	31	0.002	F (31, 181) = 2.286	P=0.0004
treatment	0.03839	1	0.038	F (1, 181) = 48.57	P<0.0001
**Dry Weight**					
Interaction	2.54	31	0.082	F (31, 234) = 0.6896	P=0.8922
genotype	14.34	31	0.463	F (31, 234) = 3.892	P<0.0001
treatment	0.2005	1	0.201	F (1, 234) = 1.687	P=0.1952
**6MSO**					
Interaction	0.006081	30	2E-04	F (30, 150) = 0.5912	P=0.9537
genotype	0.03209	30	0.001	F (30, 150) = 3.120	P<0.0001
treatment	0.000201	1	2E-04	F (1, 150) = 0.5860	P=0.4452
**I3M**					
Interaction	0.02648	31	9E-04	F (31, 165) = 0.7404	P=0.8370
genotype	0.06477	31	0.002	F (31, 165) = 1.811	P=0.0095
treatment	0.004573	1	0.005	F (1, 165) = 3.963	P=0.0482
**8MSO**					
Interaction	0.1715	31	0.006	F (31, 181) = 0.6081	P=0.9490

genotype	2.066	31	0.067	$F (31, 181) = 7.328$	$P<0.0001$
treatment	0.01331	1	0.013	$F (1, 181) = 1.464$	$P=0.2279$
