# Supplementary material for: Breeding indoor watercress for enhanced crop biofortification: harnessing natural variation of wild germplasm
Source: Front Plant Sci. 2025 Jun 20;16:1602171. doi: 10.3389/fpls.2025.1602171 (PMC12226469; doi:10.3389/fpls.2025.1602171)
Supplement: Supplementary file 5 [file Table5.pdf]

Supplementary Table 2 An accession table describing all genotypes in the manuscript. The first column shows accession number, and the second column indicates location by country. Varietal name is given for commercial lines, and country name is given for all wild-collected and grower- supplied lines.

Accession	Location
WX0003	United Kingdom
WX0006	United Kingdom
WX0007	United Kingdom
WX0009	Warwick Gene Bank
WX0011	Warwick Gene Bank
WX0013	Warwick Gene Bank
WX0015	Warwick Gene Bank
WX0018	Warwick Gene Bank
WX0020	Italy
WX0021	Italy
WX0023	USA
WX0027	Agrião Da Água Folha Larga
WX0028	Royal Sluis
WX0029	Daehnfeldt Denmark
WX0031	Cresson de Fontaine
WX0032	Brunnenkresse
WX0033	New Zealand
WX0034	Warwick Gene Bank
WX0036	Warwick Gene Bank
WX0037	Warwick Gene Bank

WX0038	Warwick Gene Bank
WX0039	UK
WX0043	Thompson & Morgan
WX0044	Mr Fothergills
WX0048	Cook's Garden
WX0050.2	UK
WX0051	UK
WX0053	UK
WX0054	UK
WX0057	USA
WX0058	China
WX0059	UK (commercial control)
